# Analysis of an Ebola virus disease survivor whose host and viral markers were predictive of death indicates the effectiveness of medical countermeasures and supportive care

**DOI:** 10.1186/s13073-020-00811-9

**Published:** 2021-01-11

**Authors:** Andrew Bosworth, Natasha Y. Rickett, Xiaofeng Dong, Lisa F. P. Ng, Isabel García-Dorival, David A. Matthews, Tom Fletcher, Michael Jacobs, Emma C. Thomson, Miles W. Carroll, Julian A. Hiscox

**Affiliations:** 1 Public Health England, Manor Farm Road, Porton Down, Salisbury, UK; 2 Clinical Virology, University Hospitals Birmingham NHS Foundation Trust, Birmingham, UK; 3 Health Protection Research Unit in Emerging and Zoonotic Infections, National Institute for Health Research, Liverpool, UK; 4 Institute of Infection, Veterinary and Ecological Sciences, University of Liverpool, Liverpool, UK; 5 Infectious Disease Horizontal Technology Centre (ID HTC), A*STAR, Singapore, Singapore; 6 School of Cellular and Molecular Medicine, University of Bristol, Bristol, UK; 7 Liverpool School of Tropical Medicine, Liverpool, UK; 8 Department of Infection, Royal Free London NHS Foundation Trust, London, UK; 9 MRC-University of Glasgow Centre for Virus Research, Glasgow, UK; 10 Nufield Department of Medicine, Wellcome Trust Centre for Human Genetics, University of Oxford, Oxford, UK

## Abstract

**Background:**

Ebola virus disease (EVD) is an often-fatal infection where the effectiveness of medical countermeasures is uncertain. During the West African outbreak (2013–2016), several patients were treated with different types of anti-viral therapies including monoclonal antibody-based cocktails that had the potential to neutralise Ebola virus (EBOV). However, at the time, the efficacy of these therapies was uncertain. Given the scale of the outbreak, several clinical phenotypes came to the forefront including the ability of the same virus to cause recrudescence in the same patient—perhaps through persisting in immune privileged sites. Several key questions remained including establishing if monoclonal antibody therapy was effective in humans with severe EVD, whether virus escape mutants were selected during treatment, and what is the potential mechanism(s) of persistence. This was made possible through longitudinal samples taken from a UK patient with EVD.

**Methods:**

Several different sample types, plasma and cerebrospinal fluid, were collected and sequenced using Illumina-based RNAseq. Sequence reads were mapped both to EBOV and the human genome and differential gene expression analysis used to identify changes in the abundance of gene transcripts as infection progressed. Digital Cell Quantitation analysis was used to predict the immune phenotype in samples derived from blood.

**Results:**

The findings were compared to equivalent data from West African patients. The study found that both virus and host markers were predictive of a fatal outcome. This suggested that the extensive supportive care, and most likely the application of the medical countermeasure ZMab (a monoclonal antibody cocktail), contributed to survival of the UK patient. The switch from progression to a ‘fatal’ outcome to a ‘survival’ outcome could be seen in both the viral and host markers. The UK patient also suffered a recrudescence infection 10 months after the initial infection. Analysis of the sequencing data indicated that the virus entered a period of reduced or minimal replication, rather than other potential mechanisms of persistence—such as defective interfering genomes.

**Conclusions:**

The data showed that comprehensive supportive care and the application of medical countermeasures are worth pursuing despite an initial unfavourable prognosis.

## Background

Ebola virus disease (EVD) has a high case fatality rate and is caused by infection of humans with Ebola virus (EBOV) [1]. Once confined to isolated outbreaks, West Africa witnessed the largest ever EBOV outbreak between 2013 and 2016 [2] and a large outbreak then occurred in the Democratic Republic of Congo [3]. The outcome of infection (death/survival) in EVD may be influenced by several factors including viral load [4, 5], the host response [6, 7] and the presence of other infections at the time of acute symptoms [8]. Generally, at the time of diagnosis, for the West African outbreak, patients with higher viral loads (as measured by RT-qPCR) had a poorer prognosis than patients with lower viral loads [5]. RNA sequencing of blood samples taken by the European Mobile Laboratory (EMLab) from acute patients at the time of presentation to a treatment centre during the West African outbreak indicated that the blood transcriptome was different between individuals in the acute phase who went on to have a fatal infection or survived [6, 7]. This included stronger upregulation of interferon signalling and acute phase responses in patients progressing to a fatal infection and increased NK-cell populations in patients who went on to survive infection. During the West African outbreak, several medical countermeasures were used and evaluated both in Africa and on repatriated health care works including favipiravir, convalescent plasma and monoclonal antibodies targeted against the EBOV glycoprotein (GP) [9–11]. Whilst the presence of EBOV in seminal fluid was described in 1977 [12] and shown to persist after non-detectable amounts were found in the blood [12], the West African outbreak illustrated that a number of patients had recrudescent infections. This often correlated with persistence of the virus in what can be described as immune-privileged sites including the testes [13] and the eye [14]. Analysis of EBOV evolution rates in semen suggested a mechanism of persistence rather than latency and indicated that EBOV replication continued during convalescence [15].

Thus, several key questions remained including establishing if monoclonal antibody therapy was effective in humans with severe EVD, whether virus escape mutants were selected during treatment, and what is the potential mechanism(s) of persistence. Such a study was made possible in a healthcare worker in the UK with EVD (referred to as UK2), who had recently returned from Sierra Leone and who developed relapse of EVD in association with meningoencephalitis 10 months after initial presentation [16]. Longitudinal and recrudescence blood, plasma, and cerebral spinal fluid samples were taken from UK2; RNA was extracted and sequenced. This provided a unique opportunity to study both EBOV population genetics and the host response over the course of EVD and to probe the effectiveness of antibody-based interventions given as part of extensive supportive care. The peak viral load in UK2 during the early course of infection and together with host markers was predictive of a fatal outcome when compared to equivalent Guinean patients. Our study found little change in overall EBOV consensus sequence, or at a minor variant level despite the application of selection pressure (in the form of antibody-mediated therapy) or between initial infection and recrudescence 10 months later, suggesting a phase of reduced or low level replication, with concomitant undetectable viral load. Changes in the blood transcriptome during EBOV infection in UK2 demonstrated that many of the markers for inflammation and the innate response were much more abundant than that of patients in West Africa, at a similar time point in infection, who went on to have a fatal infection and far greater than those who survived in this setting. This study provides molecular data to support the observation that the extensive care given to UK2 tipped the balance to survival [17]. The unusual situation of a patient recovering from an infection associated with very high viral load (that without high level critical care would almost certainly have resulted in death) could also prove useful for comparison with fatal EBOV challenge experiments in non-human primate models for the purposes of evaluating medical countermeasures.

## Methods

### Sampling

Samples were collected as part of diagnostic testing process and retained for research purposes with patient consent. Samples were tested in accordance with consent and ethical approval. All blood specimens collected for RT-qPCR in this study were collected into EDTA blood collection tubes. CSF was collected directly into plastic tubes without buffer. Blood and CSF was processed for RNA extraction immediately on arrival into the diagnostic testing laboratory and residual RNA used in this analysis.

### RT-qPCR analysis of viral RNA

For extraction and purification of viral RNA, the QiaAMP Viral RNA Mini Kit (Qiagen) was used. This kit was validated as a suitable method for extraction of viruses by diagnostic clinical laboratories, and the ability of the contained Buffer AVL to neutralise Ebola virus has supporting evidence in the literature [18]. Initial samples prior to confirmed diagnosis of EVD were handled at ACDP (Advisory Committee for Dangerous Pathogens) Containment Level 3. Subsequent specimens were handled in ACDP Containment Level 4 facilities. Inactivation was performed by addition of 140 μl plasma into 560 μl Buffer AVL; after 10 min incubation at room temperature, 1:1 volume of 100% molecular grade Ethanol was added (Sigma-Aldrich) and incubated for a further 10 min. After inactivation specimens were surface disinfected by 10% sodium hypochlorite and transferred to Containment Level 2 for extraction and purification. Viral RNA was quantified using the Nanodrop 2000 (Thermo Scientific) or Qubit (ABi; Thermo Scientific). The standard Nanodrop 2000 assay for RNA concentration estimation was performed and ratios at 260/280 nm and 260/230 nm calculated to ascertain purity. Samples below an expected value of 1.8 were retested and, if persistently below this level, were re-extracted; no samples used in this project failed this basic quality check. For concentration estimation by Qubit assay, the Broad Range RNA kit (ABi; Thermo Scientific) was used. A synthetic control was designed using sequence from the region targeted by the RT-qPCR assay used in this project to use as control template [19]. This synthetic oligonucleotide sequence was cloned into the pMX-01 vector under the control of a T7 promoter (GENEArt; Thermo Scientific) and sequence verified by Sanger sequencing at GENEArt (Thermo Scientific) and Public Health England’s Sequencing Service, Colindale, London, UK. Quantitative real-time reverse transcription polymerase chain reaction (RT-qPCR) was used to quantify the abundance of viral genome in study materials. Rather than designing a new assay, an assay with established effectiveness and with validation data supporting usage was selected. This assay has been validated for clinical diagnostic use in the Rare & Imported Pathogens Laboratory, part of Public Health England, UK, and utilised in examining samples from patients with EVD [19].

The RT-qPCR primers were designed as described for EBOV targeting the glycoprotein (GP) gene sequence, including MGB probe design (Thermo Scientific). Forward primer (900 nM), 900 nM Reverse primer and 250 nM Probe were added to each reaction. Sequences of the primers and probe were Forward: 5′-TTT TCA ATC CTC AAC CGT AAG GC-3′, Reverse: 5′-CAG TCC GGT CCC AGA ATG TG-3′, and Probe: 6FAM-CAT GTG CCG CCC CAT CGC TGC-BHQ-3′. The TaqMAN Fast Virus kit was used for reverse transcription and PCR (Thermo Scientific), and water used in the reaction was nuclease free water (Qiagen).

### Sequencing and bioinformatics analysis

Samples from the patient from the initial infection or recrudescence were either sequenced on MiSeq or HiSeq platforms as described previously [8, 16, 20]. Briefly, RNA extraction from plasma and CSF was carried out in Containment Level 3 on the easyMag platform according to the manufacturer’s instructions, or on further samples from the initial infection by PHE at Containment Level 4. CSF eluate was treated with RNase-free DNase I (Ambion), purified with RNAClean XP magnetic beads (Beckman Coulter) and eluted into 11 μl of water. Plasma eluate was concentrated using magnetic beads as indicated above, in the absence of DNase I treatment. Samples were reverse transcribed using Superscript III (Invitrogen) followed by dsDNA synthesis with NEB Next(r) mRNA Second Strand Synthesis Module (NEB). Libraries were prepared using a KAPA DNA Library Preparation Kit (KAPA Biosystems), following a modified protocol as previously described. Resulting libraries were quantified using a Qubit 3.0 fluorometer (Invitrogen) and their size determined using a 2200 TapeStation (Agilent). Libraries were pooled in equimolar concentrations and sequenced on the MiSeq and NextSeq Illumina platforms.

In this analysis, a human genome assembly GRCh38 with its gene structures (release-91) from Ensembl and an EBOV genome (GenBank sequence accession: KY426690) were used as references. Tophat2 v2.1.1 [21] was used to map RNAseq reads to the human reference and Cufflinks v2.2.1 [22] to determine normalised human gene expression levels (FPKM). Hisat2 v2.1.0 [23] was used to map the trimmed reads on the human reference genome assembly with default setting.

FPKM calculated using Cufflinks was used as input data into Digital Cell Quantitation, which uses transcriptomic patterns to infer the predicted activity of immune cell types. The unmapped reads were extracted by bam2fastq (v1.1.0) and then mapped on the EBOV reference genome using Bowtie2 v2.3.5.1 [23] by setting the option to parameters “--local”, followed by Sam file to Bam file conversion, sorting, and removal of the reads with a mapping quality score below 11 using SAMtools v1.9 [24]. After that, the PCR and optical duplicate reads in the bam files were discarded using the MarkDuplicates in the Picard toolkit v2.18.25 (http://broadinstitute.github.io/picard/) with the option of “REMOVE_DUPLICATES=true”. The resultant Bam file was processed by Quasirecomb v1.2 [25] to generate a phred-weighted table of nucleotide frequencies which were parsed with a custom perl script to generate a consensus genome sequence as our previous description [4]. The consensus genome was then used as a template in the second round of mapping to generate the final consensus genome sequences and corresponding phred-weighted table of nucleotide frequencies. yn00 in the PAML package in the R environment was utilised to calculate rate of nucleotide substitution within the patient and compare this with published rate of substitution determined through analysis of multiple patients across transmission events. Consensus genome sequences (Supplementary data 1) and a phred-weighted table of nucleotide frequencies (Supplementary data 1) were obtained for each sample. These nucleotide frequencies were used for comparison of minor variation. These consensus genome sequences were aligned with Mafft v 7.402 [26]. The aligned genomes were partitioned into four sets of sites: 1st, 2nd and 3rd codon positions of the protein-coding regions and the noncoding intergenic regions. PartitionFinder v2.1.1 [27] was then used to find the best-fit substitution model and distribution of rates for each set. Four Bayesian nucleotide divergence tree for these sets was then constructed using MrBayes v3.2.6 [28], a Bayesian analysis tool with their best-fit substitution model and distribution of rates. The final consensus genome sequences were also used as reference to identify DI events by running the DI-tector v0.6 [29] programme with default setting.

## Results

Studies describing EVD over the course of an infection in humans under controlled conditions are rare. Generally, these have made use of laboratory accidents [12] or healthcare and related workers who were given extensive supportive care, and treated with experimental medical countermeasures [30]. Currently, none of the medical countermeasures for Ebola virus are of absolute proven benefit, although some hold promise [10, 11]. The analysis of longitudinal samples provides greater insight into EVD, and where given, the potential role of medical countermeasures in facilitating survival.

### Overview of UK2 upon presentation with and during EVD

In December 2014, a healthcare worker returned to the UK from West Africa with acute febrile illness. On presentation at hospital (referred to as day 1), the patient had a markedly elevated temperature of 39.3 °C and was haemodynamically unstable but responded rapidly to rapid intravenous fluids and antibiotic therapy. RT-qPCR for EBOV genome was performed by the Royal Infirmary of Edinburgh Clinical Virology Department in conjunction with the Rare & Imported Pathogens Laboratory (days 1–2) and Virus Reference Department, Colindale (days 3–28) (part of PHE). The patient’s initial sample tested positive for EBOV RNA, and the patient was transferred to the high-level isolation unit at the Royal Free Hospital, London, UK, on the 2nd day after admission (referred to in this study as day 2). To ensure consistency, all samples used in this study were retested and EBOV quantified using the Trombley RT-qPCR assay that targets a region within the GP coding sequence.

A Ct value of 23.9 in the GP gene qRT-PCR assay was recorded at presentation (day 1). On day 2, this changed to Ct 21.2 in the GP gene assay. The patient was treated with one oral dose of Brincidofovir (200 mg) (an experimental antiviral drug) and two doses of convalescent plasma (300 ml) on consecutive days, obtained from a survivor of EVD. By day 5, symptoms worsened, and the patient developed severe watery diarrhoea and type 1 respiratory failure. The patient also developed erythroderma, mucositis and agitation, but did not develop overtly haemorrhagic manifestations.

The highest viral load was recorded on day 6 (Ct 14.3), and the severity of the patient’s illness approximately correlated with viral load. The viral load data from this patient was compared with diagnostic findings from European Mobile Laboratory in the 2013–2016 West African outbreak [5]. West African patients in the outbreak with Ct of ~ 14, and treated in West Africa, all had a fatal outcome. This illustrated the severity of illness for UK2, and on this basis, the patient would have been predicted not to survive EVD.

The patient was administered ZMAb (50 mg/kg) by infusion on day 5 and day 8. After day 12 of the illness, the viral load began to decrease. By day 21, the patient had developed a pronounced thrombocytosis (peaking at 1726 × 10^9^/L; normal range 150–400) and was discharged on low-molecular-weight aspirin and heparin [31] following two consecutive negative RT-qPCR further on days 25 and 28 of illness, in accordance with general WHO advice. The overall course of illness was very similar to the clinical findings of outbreaks caused by other variants of EBOV. However, 10 months following this illness, the patient was readmitted with a previously undescribed presentation of relapse of EVD in association with the presence of virus in both plasma and cerebral spinal fluid (CSF) [16].

**Fig. 1:**
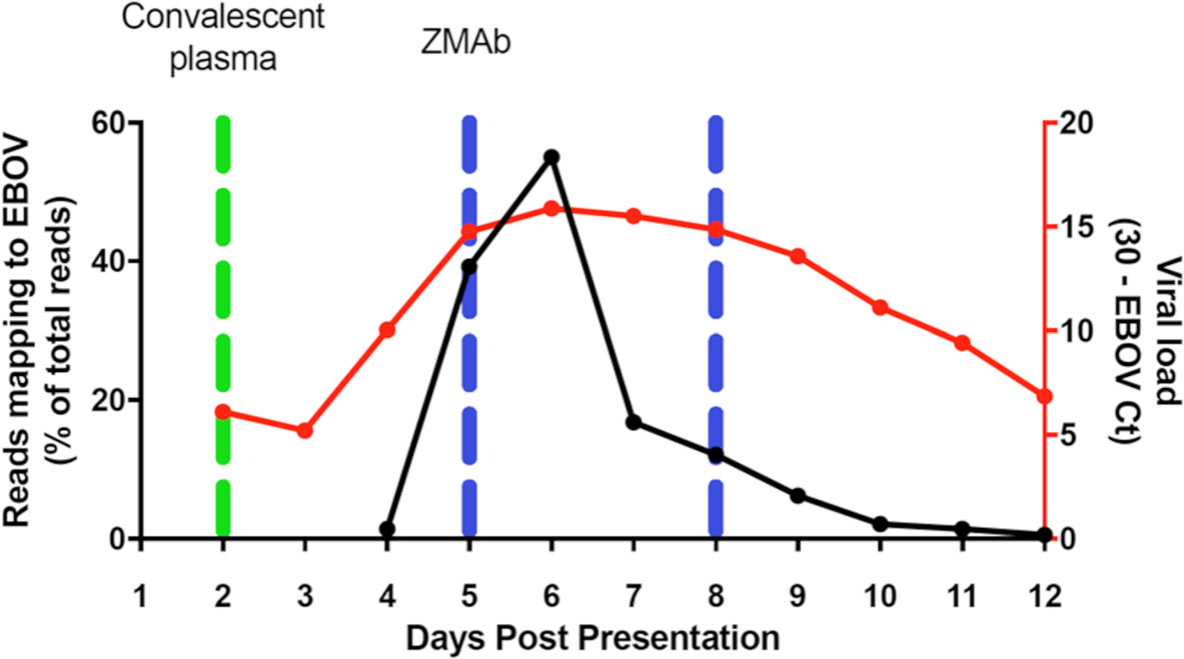
Time-course showing viral load as measured by RT-qPCR of the GP gene of EBOV in samples collected from a UK case of EVD. The *X*-axis displays the days post symptoms. The right-hand *y*-axis displays the relative viral genome abundance (calculated by PCR maximum cT (30) − sample cT to calculate the proportional inverse value). The left-hand *y*-axis displays RNAseq data from the blood samples as reads mapping to the EBOV genome as a proportion of total reads. The peak of viremia is discernible at day 6

To investigate virus evolution and the host response to EBOV over the course of the primary infection and during recrudescence, longitudinal analysis using RNAseq was carried out using available samples and compared to equivalent patients from West Africa (Table 1). These included a plasma sample sequenced upon initial admission (day 1), blood samples taken over the acute phase between days 4 and 12, and during recrudescence, a plasma and CSF samples. These latter samples were from the same lumbar puncture, but separate aliquots sent to independent laboratories who received them on different days. Sequence reads were mapped to the EBOV genome and the human genome (Table 1). The sequence read depth across the EBOV genome was approximately equivalent for the different samples suggesting that viral genomic RNA had been sequenced rather than EBOV mRNA (Fig. 1), which would have been associated with a higher proportion of reads mapping to the genes located towards the 3′ end of the negative strand genome [32].

**Table 1:** Sequencing reads across patient timeline for UK2 and also comparative data from selected EMLab patients (IDs 413, 753, 578, 462 and 351).

	Plasma	Blood								
	Day 1	Day 4	Day 5	Day 6	Day 7	Day 8	Day 9	Day 10	Day 11	Day 12
Total reads	13,431,416	3,833,134	2,645,578	2,177,628	5,431,570	5,705,462	1,864,628	3,049,104	1,481,988	4,342,832
Reads mapped to EBOV	595 (0.00%)	54,856 (1.43%)	1,037,421 (39.21%)	1,198,007 (55.01%)	914,490 (16.84%)	692,250 (12.13%)	116,405 (6.24%)	65,188 (2.14%)	21,352 (1.44%)	25,429 (0.59%)
Reads mapped to human	12,555,014 (93.47%)	1,436,018 (37.5%)	862,860 (32.6%)	519,467 (23.9%)	3,116,172 (57.4%)	3,487,829 (61.1%)	1,239,462 (66.5%)	1,980,054 (64.9%)	916,534 (61.8%)	2,736,038 (63%)
	Plasma Month 10	CSF Month 10 071015	CSF Month 10 081015		EMLab Patient ID	413	753	578	462	351
Total reads	14,377,682	11,626,808	12,774,412		Total reads	56,565,350	6,398,890	78,423,238	47,990,570	75,648,710
Reads mapped to EBOV	180 (0.00%)	9227 (0.08%)	10,200 (0.08%)		Reads mapped to EBOV	20,471,809 (36.19%)	1,099,593 (17.18%)	7,544,790 (9.62%)	4,469,920 (9.31%)	5,525,548 (7.30%)
Reads mapped to human	13,590,746 (94.53%)	11,073,317 (95.24%)	12,164,725 (95.23%)		Reads mapped to human	33,939,691 (60.00%)	4,853,256 (75.85%)	69,922,793 (89.16%)	42,888,768 (89.37%)	68,391,308 (90.41%)

### Analysis of viral genome abundance by RNA-seq in UK2 with time post-admission suggested a viral load associated with a fatal outcome at the early stages of infection

The sequencing data from the blood samples (Table 1) also provided an independent comparison of the relative amount of EBOV between each day post admission using the number of sequence reads mapped to the viral genome and clarified ambiguity with the RT-qPCR-based assays. Analysis of diagnostic leftover plasma/blood samples taken from patients during the West African outbreak in Guinea showed a close correlation at admission between viral load as determined by RT-qPCR, genome abundance determined by the number of mapped reads and outcome (survival/death) [4]. Therefore, for each sequenced sample for UK2, the proportion of reads mapping to the EBOV genome was compared to the total number of reads (Table 1). This value was then compared to the viral load data—derived from RT-qPCR analysis of blood samples (Fig. 1). This comparison revealed that where data for both viral load and reads mapping to the EBOV genome were available, there was a close correlation between both measurements. Both viral load and percentage reads mapping to the EBOV genome peaked on day 6. The percentage of reads mapping to the EBOV genome illustrated a sharp increase in EBOV over a 2-day period from day 4 (1.43%) to day 6 (55.01%). After the infusion of ZMAb on day 5, the reads mapping to the viral genome dropped between day 6 and day 7. However, not until days 11/12 did the percentage of reads mapping to the EBOV genome decrease below the day 4 value. Also, for day 6, most sequence reads mapped to the EBOV genome (Table 1).

Analysis of RNAseq data between UK2 and gender matched data from similar blood samples from adult Guinean patients with EVD at the time of admission (Table 1) allowed comparative quantification of viral loads. The samples from Guinea were taken on average 6 days post symptom onset and therefore are approximately equivalent to UK2 at the early stage of infection. Patient 413 (Table 1) had the largest proportion of sequence reads mapping to EBOV, yet this was less than UK2 at day 6. This provided further evidence for the severity of this EBOV infection in the UK2 patient, and based on this, the prediction of a fatal outcome in UK2.

### Measurement of host biomarkers in UK2 and comparison to acutely ill Guinean patients were indicative of a fatal outcome

Analysis of the blood transcriptome in patients with EVD can provide detailed information on the host response to EBOV infection [6, 7]. The RNAseq data provided a readout on what host RNAs increased and decreased in abundance in patients with EVD compared to patients without EVD. These changes in abundance may have been caused by differential gene expression and/or an influx or efflux of different cell types from the blood. In this study, we made use of the sequencing data to map reads to the human genome (Table 1). For six of the samples, from day 7 to day 12, the number of reads that could be mapped to the human genome was around 60%. For the other days, a lower number of reads mapped to the human genome, reflecting the very high viral load in UK2, and dominance of EBOV RNA in the blood transcriptome. The abundance of transcripts was inspected across the time course to ensure that no major variation in either library preparation on sequencing run introduced bias. In many cases, the abundance of transcripts in the day 4 samples was higher than any of the other samples; therefore, the dataset from this day was treated with caution. Values from this day are included for completeness in the analysis of differential gene expression but should not be considered definitive.

**Fig. 2:**
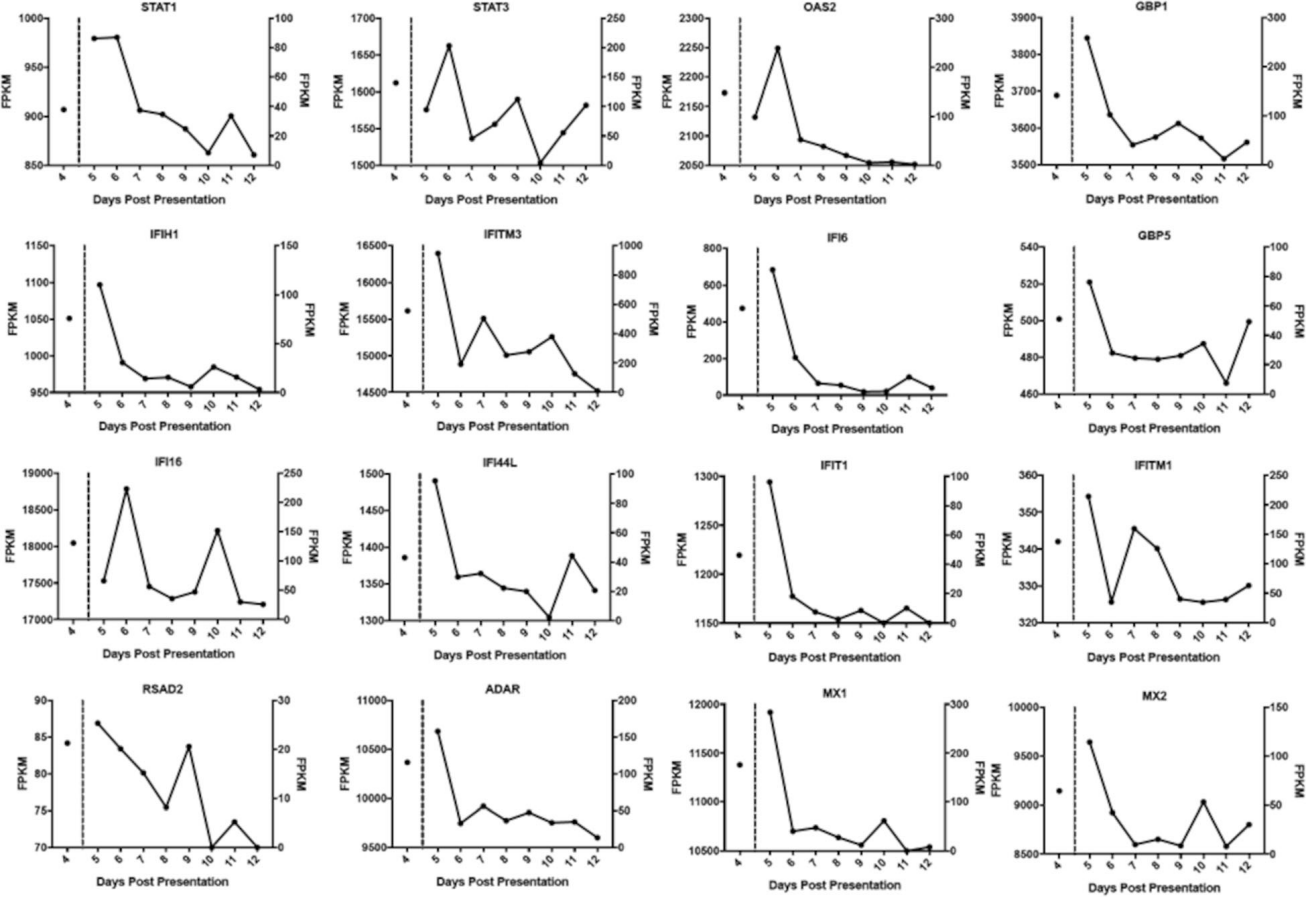
Transcript abundance data (FPKM) displayed for 16 genes characterised as known interferon stimulated genes (ISG). The transcript abundance for each of the genes correlates well with abundances of viral RNA and they decrease over time. Transcript FPKM is shown on the *y*-axis for each gene and *x*-axis displays the days with symptoms from day 4 to day 12. The identifier for each gene is given above each chart. A zero value indicates that the transcripts was either not detected or the transcript was absent

The blood transcriptome of patients in Guinea at presentation to an Ebola virus treatment centre indicated increased levels of interferon stimulated genes (ISGs) relative to normal or convalescent controls [6]. Therefore, the abundance of ISGs in UK2 was analysed. Transcripts which were consistently detected across the longitudinal time course were identified using the Interferome v2.01 database. In the dataset, 31 potential ISGs were identified at all 9 time points (days 4–12). Transcripts encoding well-characterised interferon stimulated genes are shown in Fig. 2. FPKM values for each transcript were compared at each time point and displayed without further processing. With the general exception of the day 4 sample, the majority of gene transcript abundance peaked with maximum viral genome abundance in the samples from day 5/day 6. Reflecting data from non-human primate studies [33] and from the blood transcriptome analysis of samples from patients from Guinea [6], this suggested that the innate response was a critical aspect of the cellular response to EBOV.

**Fig. 3:**
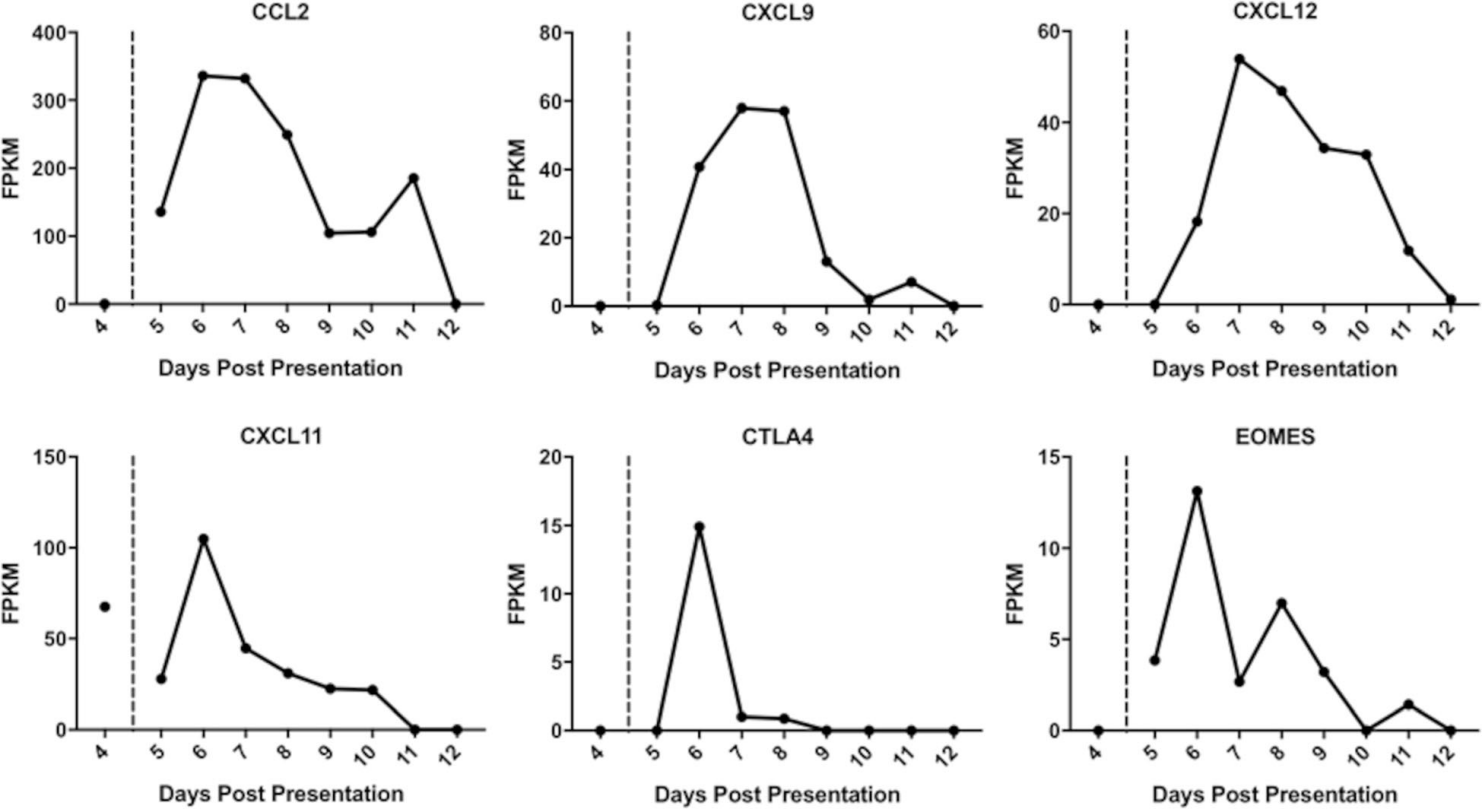
Transcript abundance data (FPKM) displayed for 6 genes characterised as involved in the immune response. The transcript abundance for each gene correlates well with abundances of viral RNA and they decrease overtime. Transcript FPKM is shown on the *y*-axis for each gene, and *x*-axis displays the days with symptoms from day 4 to day 12. The identifier for each gene is given above each chart. A zero value indicates that the transcripts was either not detected or the transcript was absent

The abundance of several host transcripts/proteins has been shown to correlate with outcome in West African patients with EVD. For example, fatal EVD has been associated with the increase in abundance of cytotoxic T lymphocyte-associated protein 4 (CTLA-4) and eomesodermin (Eomes), suggestive of defects in T cell homeostasis [34]. Where detected by sequencing, both transcripts were elevated in abundance at day 6 compared to the other days. The abundance of chemokine transcripts that could be detected, except for CXCL11 (which peaked on day 6), tended to peak on day 7. These included CXCL2, CXCL9 and CXCL12 (Fig. 3) and function to recruit T cells.

Overall, the data suggested that the IFN response was robust and to be expected in an overwhelming infection. There was no evidence of any rare immunodeficiency in innate immunity. Indeed comparison of day 6 levels to equivalent Guinean patients with a fatal outcome [6] would suggest that the innate response was generally higher than when compared to those patients who went on to survive EVD.

### Changes in the immune cell phenotype occur as infection progresses and viral load decreases

**Fig. 4:**
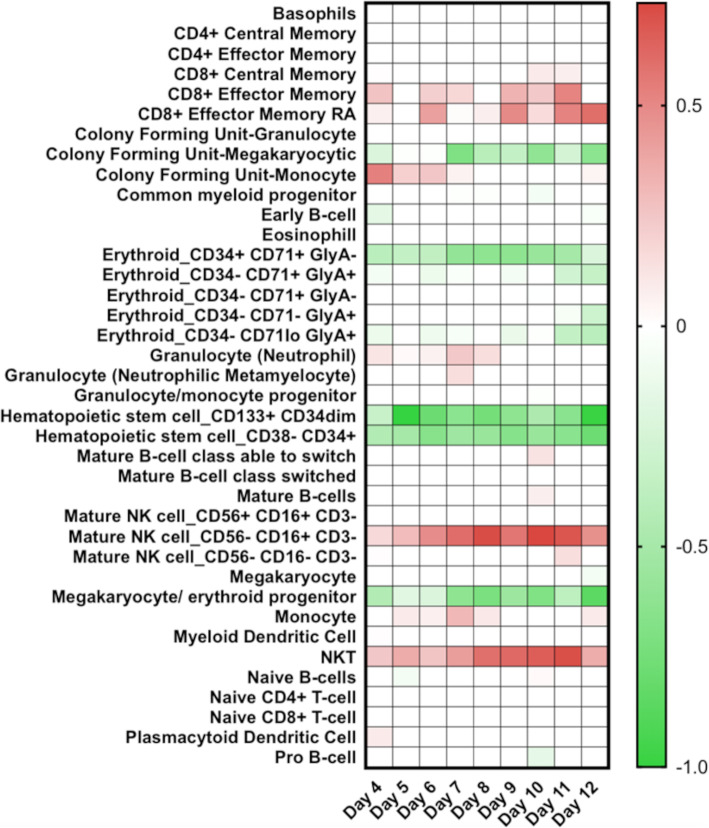
Predicted differential abundance of cell types present in the samples from UK2 at the days indicated. These are shown as relative abundance as predicted by DCQ compared to a healthy control group at each day. Within the heatmap, darker green represents a decrease in the abundance of a given cell type and darker red indicates an increase in the abundance of a specific cell type

Potential changes in the blood/plasma transcriptome can be through either changes in gene regulation such as differential expression and/or through the influx and efflux of different cell types—such as those of the immune system. Conventionally flow cytometry is used to assess these—however, this was not possible with samples from this patient. Therefore, to investigate changes in the immune cell phenotype, digital cell quantification (DCQ) [35] was used to predict which cell types may have been present at each time point by comparing the transcriptomic data to a characterised healthy control group [6]. Previously, we used this approach to interrogate similar transcriptomic data from West African patients [6]. In patients with EVD who went on to have a fatal outcome, DCQ analysis predicted that lower levels of circulating CD14+ classic monocytes would be present, which was confirmed with flow cytometry [6]. DCQ was applied to the transcriptomic data from UK2 (Fig. 4) and indicated potential changes in the abundance of a given cell type. Of note were the predicted increase in abundance of both natural killer cells (CD56− CD16+ CD3−) and natural killer T cells (NKT) cells, through alignment of the transcriptomic profile of these cell types by the DCQ process.

### Analysis of the viral genome population suggested that escape mutants to ZMab during infection and treatment were not selected or present

**Fig. 5:**
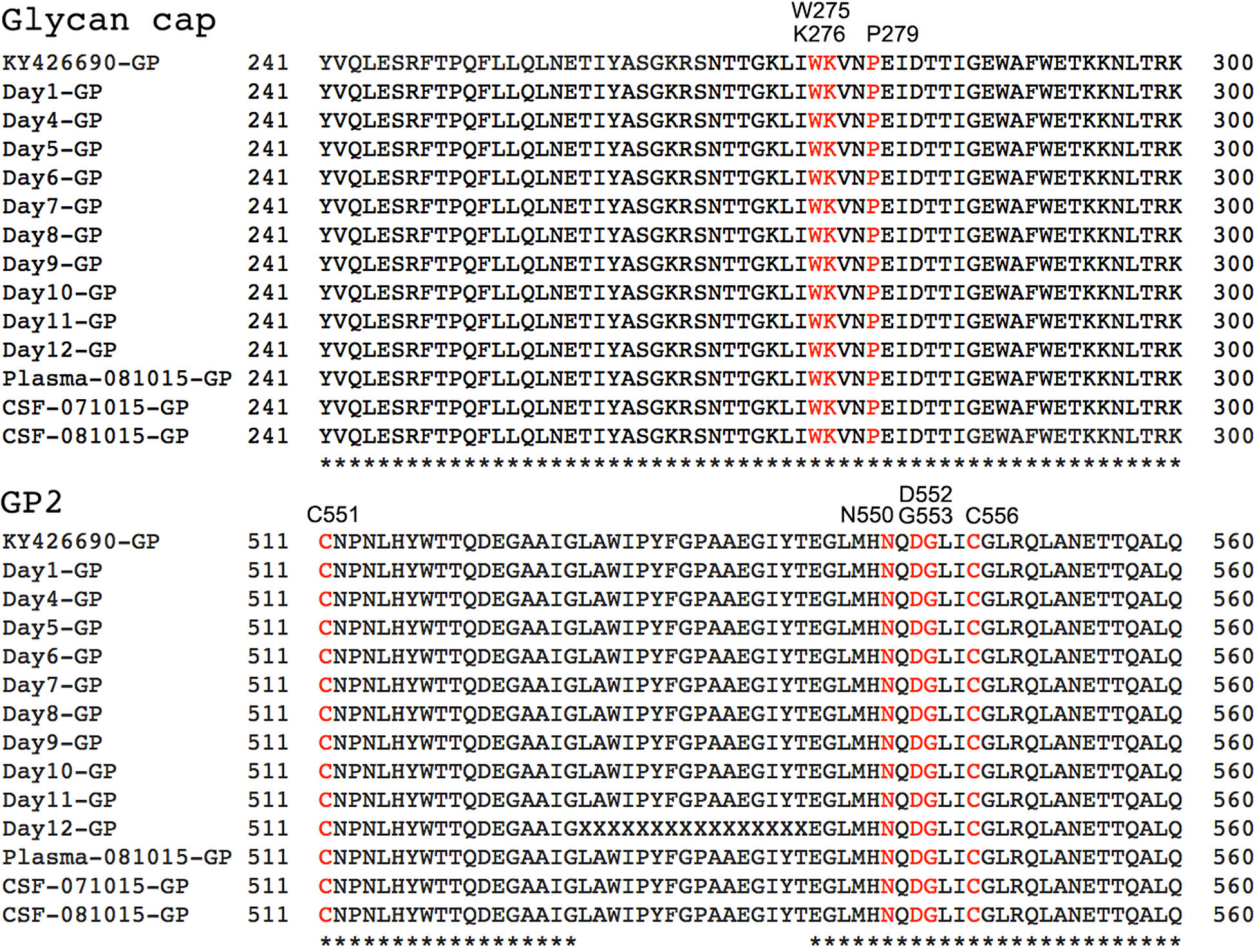
Consensus sequence in the portions of the EBOV genome corresponding to the ZMAb binding sites identified in GP. In red are amino acids targeted by the antibody cocktail that forms ZMab

UK2 was administered ZMAb at day 5 and day 8. The specific targets of ZMab are known and found in GP. Both 2G4 and 4G7 target the base of GP, between them encompassing amino acids C511, N550, D552, G553 and C556. Monoclonal antibody 1H3 targets the glycan cap and encompasses amino acids W275, K276 and P279. To investigate whether there were any changes in GP (or other EBOV proteins), EBOV consensus genomes were assembled for each time point and sample and then used to derive EBOV protein sequences. Alignment of these sequences produced a consensus sequence covering the full length of the GP gene. All the amino acids identified to be critical for targeting by ZMAb were represented in each consensus sequence (Fig. 5). This data suggested that there was no selection of antibody escape mutants to form the dominant amino acid sequence in a consensus population at the dosing schedule used.

**Fig. 6:**
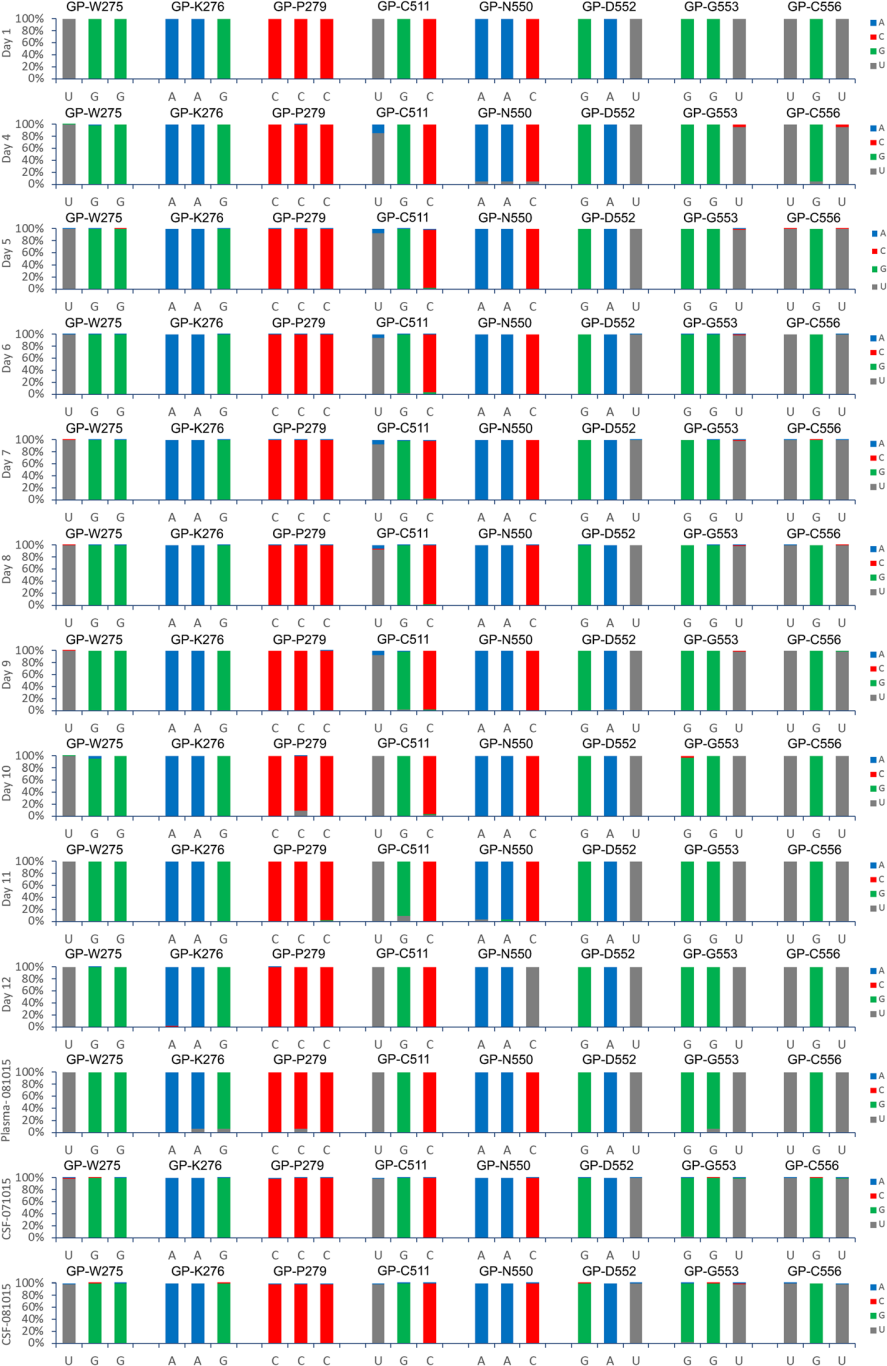
Minor variant frequencies at specified amino acid positions involved in binding the monoclonal antibodies that form ZMab in EBOV GP for the different time points post infection, including recrudescence

The application of ZMab, although not affecting the consensus EBOV genome population, may have acted as a selection pressure at the level of minor variants, e.g. changes in sequence that did not achieve a 50% threshold, and hence become consensus sequence. To investigate this, the pattern of minor variants within the EBOV genome at each time point was examined (Fig. 6). The data suggested that there was no overall large increase in minor variant frequency in any codon position and particularly at positions 1 and 2 that would have been indicative of a non-synonymous substitution and emergence of specific escape mutants to ZMab (Fig. 6).

### Analysis of virus population genetics suggested the antibody response was directed against the consensus genome population

**Fig. 7:**
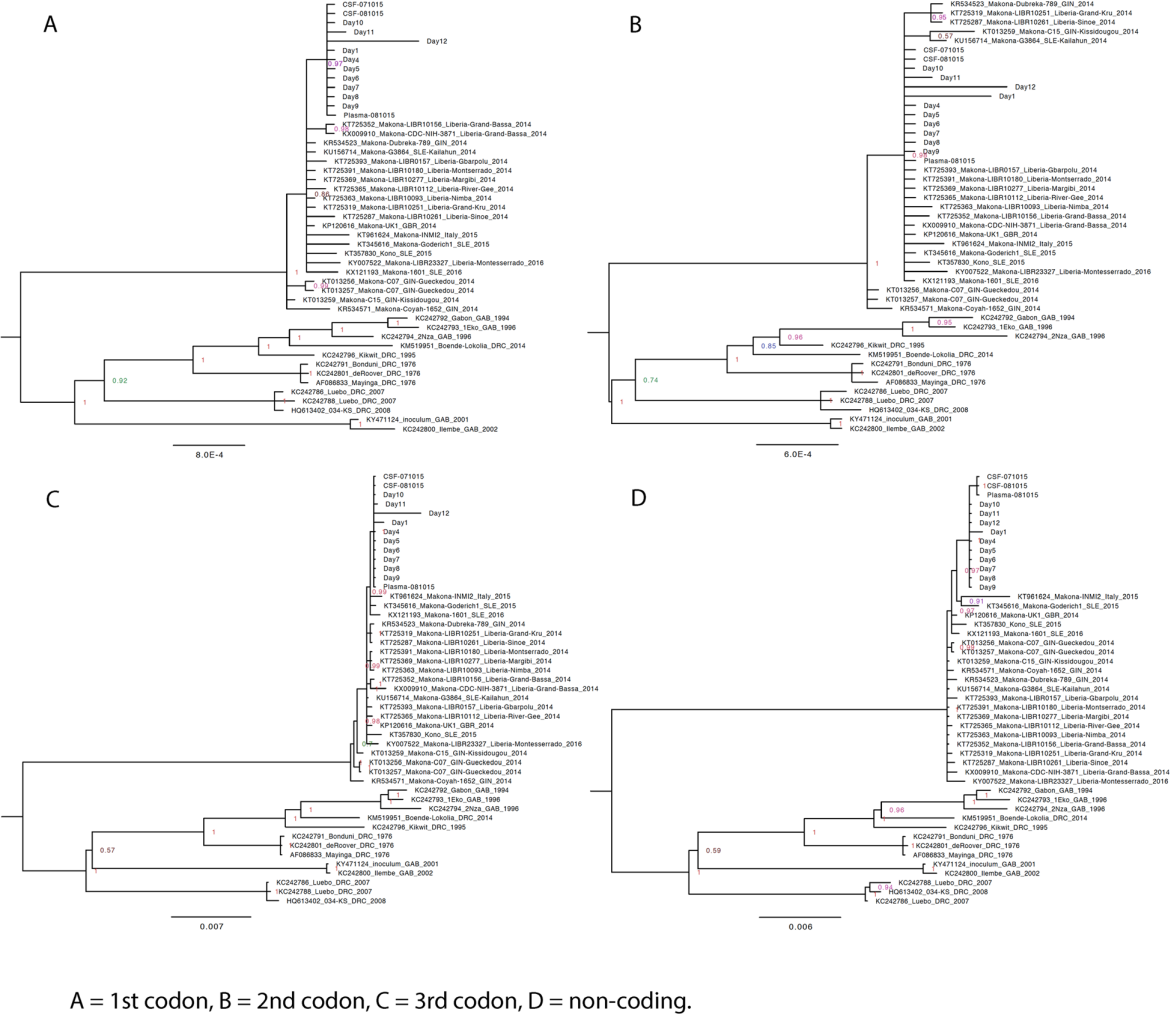
Phylogenetic analysis and comparison of EBOV sequence of the 1st, 2nd and 3rd codons and non-coding sequences in UK2 (from both the first and recrudescent infections) with EBOV genome sequences from the West African (2013–2016) and earlier outbreaks back to 1976

**Fig. 8:**
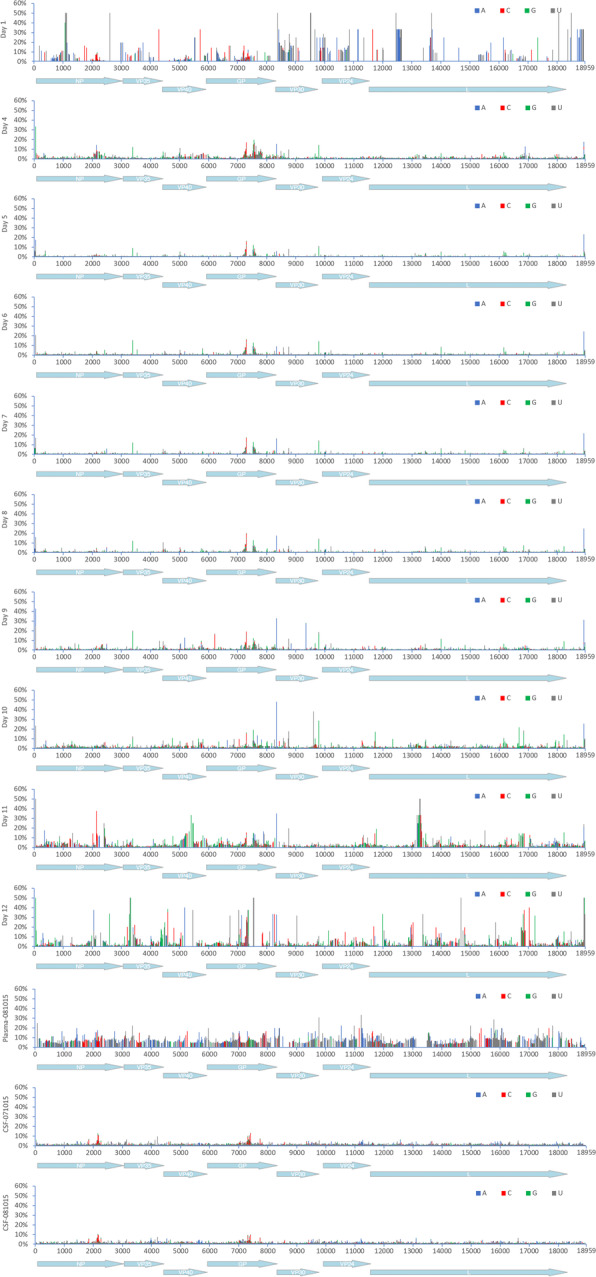
Map of minor variants across the EBOV genome for each sample (day 4 to day 12) and during recrudescence. A schematic of the position of EBOV genes along the genome is shown on the *X*-axis

**Fig. 9:**
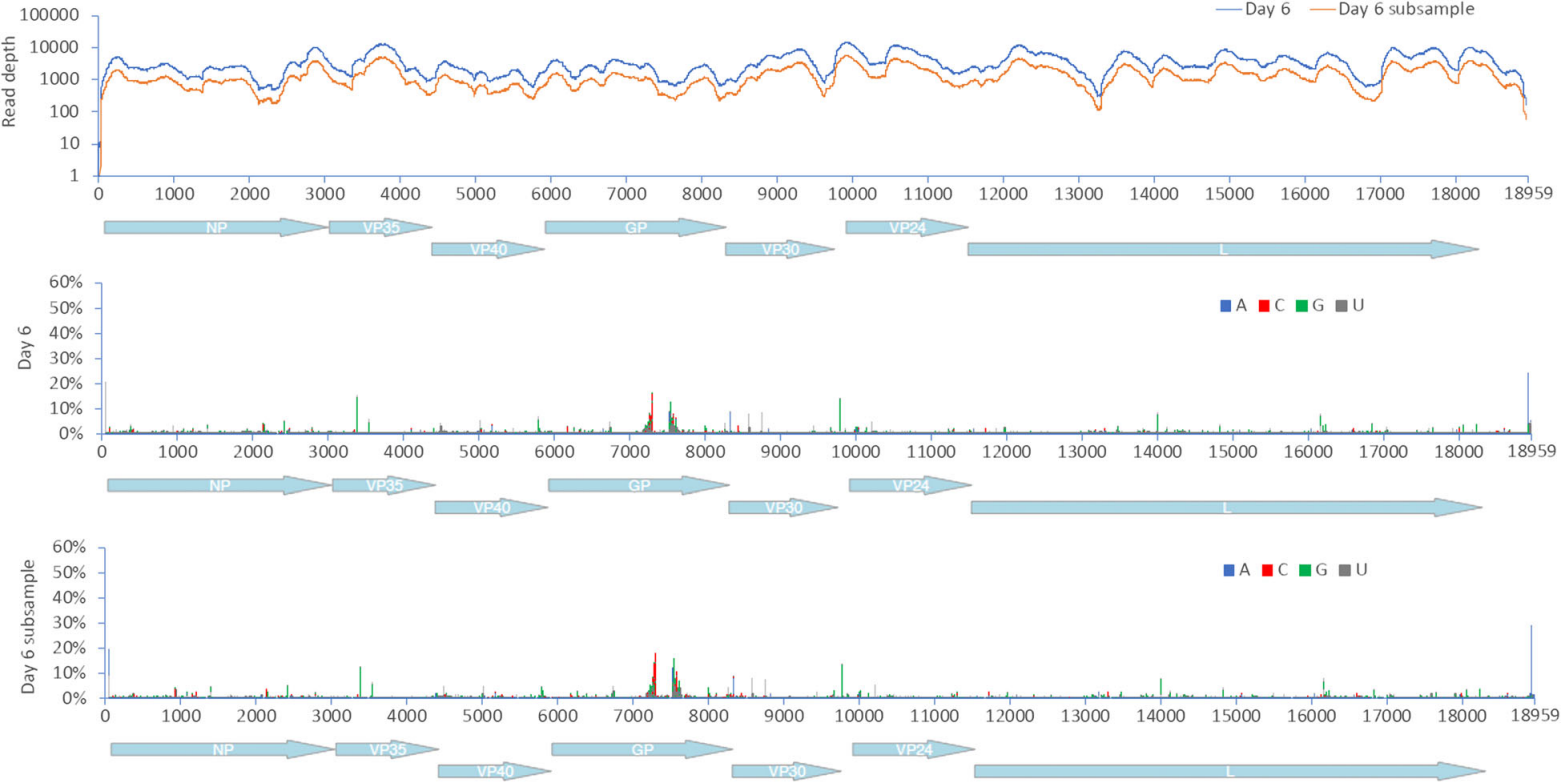
Distribution of sequence reads mapping to the EBOV genome before and after randomised sub sampling. Day 6 was chosen as a comparison point. A schematic of the position of EBOV genes along the genome is shown on the *X*-axis

During infection, there are various evolutionary pressures placed on the virus, including the innate immune system early in infection transitioning to the adaptive response and the production of neutralising antibodies some 10–12 days post-infection. Longitudinal samples were analysed to investigate EBOV population genetics in UK2. Phylogenetic analysis of the consensus nucleotide sequences of EBOV in UK2 at different sampling points indicated a lack of divergence (Fig. 7). Day 12 post admission was the most divergent sample, based on an analysis of coding sequence, with the recrudescent plasma and CSF samples being most similar to day 10/day11, suggesting that potentially prior to day 12, EBOV had migrated to the central nervous system, considered an immune privileged site. Whilst there was no overall major change in EBOV consensus sequence, variation in the EBOV genome was investigated between each sample and whether potential nucleotide variation occurred at the level of minor variants. Analysis of minor variants showed that these were frequent on day 1, and then appeared restricted on days 4 to 8 (Fig. 8). Several hypotheses could account for this. First, was the lower sequence read depths mapping to the EBOV genome, providing noisier data, although the general trend in the pattern of minor variants was similar at all days. To investigate this possibility, 62% of the reads were randomly removed from day 6 to make the read depth equivalent to the day 11 and day 12 read depths. The mapping depths were compared before and after the subsampling. This indicated the random selection had no bias and no change in the frequency of variants, suggesting that the minor variants were real phenomena (Fig. 9). Second, is that the immune therapy given (convalescent sera/monoclonal antibodies) restricted the viral genotypes. However, as described, there was no evidence of minor variants being selected in response to ZMab, and anecdotal evidence suggested that the convalescent sera used was not neutralising. Although this treatment may have placed some selective pressure on the virus through Fc mediated antiviral actions. Minor variants appeared to increase in frequency on day 9 and become more common place by day 12. This corresponded with the large decrease in viral load on these days compared to peak viral load at day 6.

### EBOV persistence may be due to a state of significantly reduced rate of viral replication rather than defective genomes

The presence of EBOV in sites described as immune privileged in both non-human primate models [36, 37] and in survivors of the 2013–2016 West African outbreak [13, 38] has been suggested as potentially containing reservoirs of virus that can initiate new infections many months after an individual has been declared free of EBOV. As described herein and previously [16], UK2 developed EVD approximately 9 months after being discharged from the first infection with EBOV. This manifested in a severe acute meningo-encephalitic illness with fever, severe headache and photophobia and bilateral VI, left pupillary-sparing partial III, left upper motor neuron VII and left VIII cranial nerve palsies and left-sided cerebellar dysfunction. A markedly higher viral load was present in CSF than plasma and MRI scanning of the brain confirmed involvement of the leptomeninges, brainstem, cranial nerves, cauda equina, conus medullaris and left cerebellar hemisphere. This fluid surrounds central nervous system sites described as immune privileged. After the case report for the recrudescent illness involving meningoencephalitis in this patient was published [16], the presence of aberrantly synthesised EBOV genomes, termed defective interfering (DI), was proposed as a potential mechanism to maintain a persistent infection of EBOV [39]. In this case, the DIs proposed, copy backs, are formed from the 5′ end of the genome, and outcompete the genome in terms of replication. Thus, from the sequence data, a greater number of sequence reads would be predicted to map to the 5′ end versus the 3′ end of the genome if this type of DI was present. Another form of DI, called a deletion DI, is created through discontinuous RNA synthesis. Alternatively, to DIs supressing or competing with virus replication, the virus may have entered a state of reduced or less efficient replication, in which case, the rate of non-synonymous versus synonymous substitutions would be lower than would predicted based on the expected rate of genome evolution from studies that include transmission. This latter hypothesis is possible because phylogenetic analysis indicated that the major evolutionary driving force in the initial infection was non-synonymous changes, whereas between the initial infection and recrudescence the predominant mutations were synonymous (Fig. 7).

**Fig. 10:**
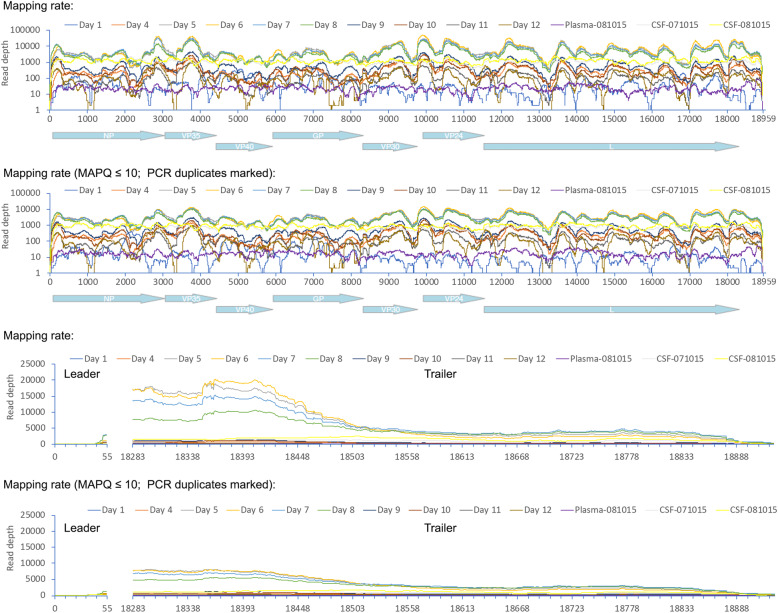
Read depth analysis of samples sequenced. A schematic of the position of EBOV genes along the genome is shown on the *X*-axis for the mapping rate, and the lower two analyses focus on the reads mapping to the 3′ and 5′ ends of the genome. Mapping rates were shown before and after filtration, i.e. the MAPQ > 10 and PCR duplicates were removed

To investigate the presence of DIs, the viral sequencing data from UK2 was analysed both for read depth at the 3′ and 5′ ends and also fusion events using the Di-tector algorithm to establish the frequency of discontinuous fusion events, which would be characteristic of deletion DIs [29]. Further, the same fusion event would be predicted to be in multiple sequential samples if the DI became established and was propagated during replication of viral RNA. The read depth analysis indicated no enrichment of sequence reads mapping to the 5′ end of the genome relative to the 3′ end suggesting that copy back DIs had not become established in any of the samples (Fig. 10).

**Fig. 11:**
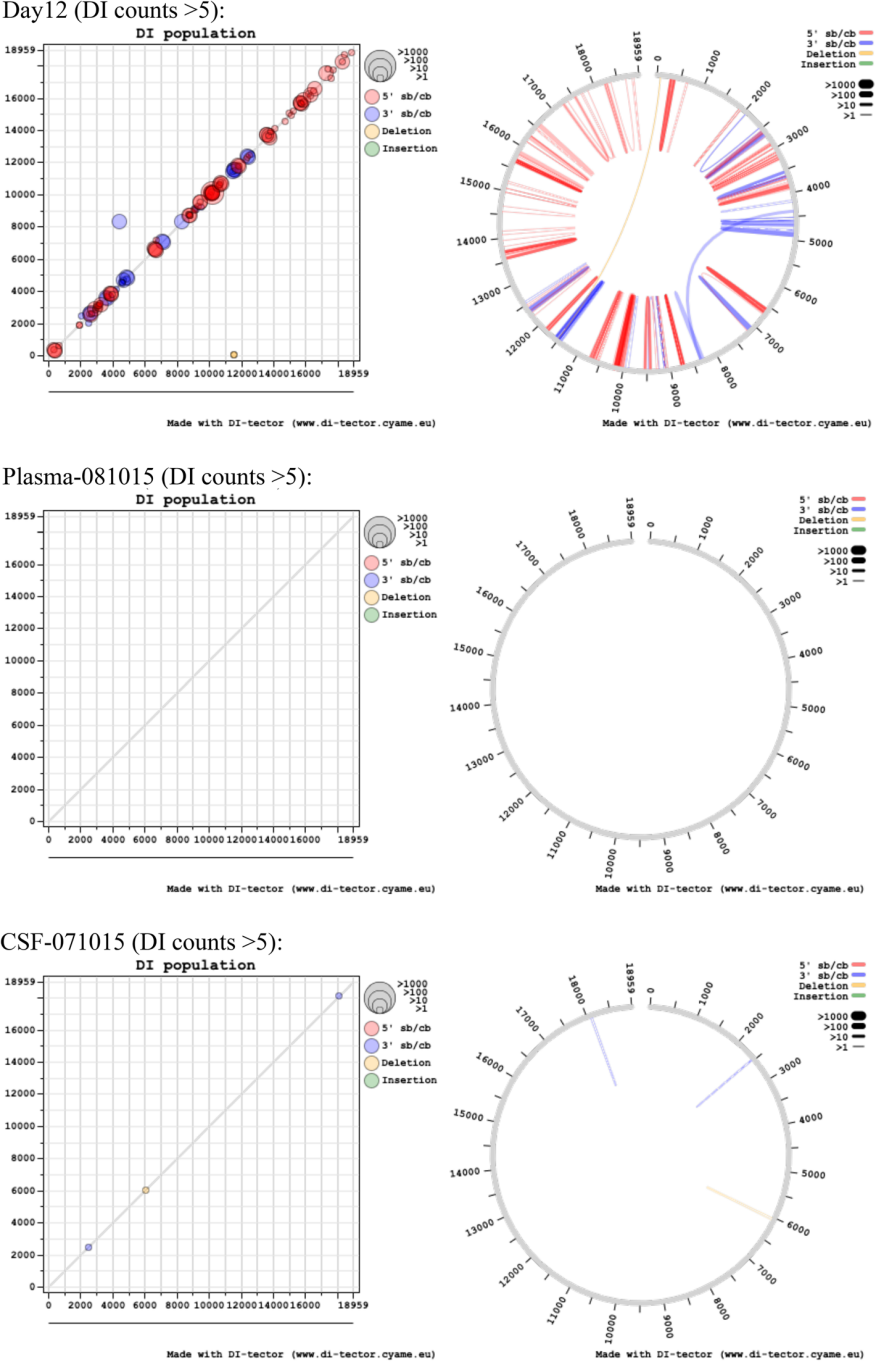
In left scatter-plot, the breakpoint site (BP) and re-initiation site (RI) positions are respectively used for “*x*” and “*y*” axis, and the closer the dot is to the diagonal of the scatter plot, the smaller the distance between BP and RI. In the right chord diagram, sequence starts at the viral genome extremity, jumps from BP to RI and terminates from RI to genome extremity again. Colour and dot/line size change according to DI genome type and frequency

Analyses of the sequence data by Di-tector indicated a diverse array of fusion events in many of the samples either within or between negative (genome) and positive (anti-genome) strands (Table 2). Mostly these were confined to short deletions of the genome. DIs can be packaged into virions and therefore spread during infection. Several the same fusion events were present in multiple days and also in the CSF and also increased with read depth from sequential days in the blood samples (Fig. 11). This suggested that potentially the same DI was being propagated. However, the reads mapping to the fusion events were never in the majority and infrequent compared to the total number of reads mapping to the EBOV genome, including in the CSF.

**Table 2:** Frequency of defective viral genomes detections

	Total DI reads	5′ DVGs	3′ DVGs	DVGs with deletion	DVGs with insertion
Day 1	83	8 (9.4%)	1 (1.2%)	28 (33.73%)	46 (55.42%)
Day 4	4096	3163 (77.22%)	815 (19.90%)	24 (0.59%)	94 (2.29%)
Day 5	68,219	55,389 (81.19%)	10,926 (16.02%)	447 (0.66%)	1457 (2.14%)
Day 6	109,650	96,162 (87.70%)	11,518 (10.50%)	498 (0.45%)	1472 (1.34%)
Day 7	90,244	71,070 (78.75%)	17,882 (19.82%)	433 (0.48%)	859 (0.95%)
Day 8	76,582	59,710 (77.97%)	16,004 (20.90%)	325 (0.42%)	543 (0.71%)
Day 9	13,202	10,145 (76.84%)	2904 (22.00%)	55 (0.42%)	98 (0.74%)
Day 10	7449	5346 (71.77%)	2074 (27.84%)	17 (0.23%)	12 (0.16%)
Day 11	2179	1678 (77.01%)	471 (21.62%)	14 (0.64%)	16 (0.73%)
Day 12	2914	2125 (72.92%)	762 (26.15%)	10 (0.34%)	17 (0.58%)
Plasma-081015	16	4 (25.00%)	11 (68.75%)	0 (0.00%)	1 (6.25%)
CSF-071015	437	154 (35.24%)	197 (45.08%)	22 (5.03%)	64 (14.65%)
CSF-081015	463	137 (29.59%)	215 (46.44%)	33 (7.13%)	78 (16.85%)

As discussed, alternatively, the virus may have entered a state of low level replication, proposed by Young et al. for other viruses [40]. Here, genome variants may be selected for in which virus replication is suppressed and may therefore evade antiviral activity and a cellular response. Previously, comparison of non-synonymous changes between EBOV in the plasma and CSF revealed only two non-coding changes, present and identical in both samples, compared with the original sequence from plasma (NCBI accession numbers KU052669 and KU052670) [16]. Herein, EBOV genome sequence from these samples was also compared to the samples taken at different days from the original infection. Given that the nucleotide substitution frequency, as calculated from data from the West African outbreak [4], was in the order of 1.42 × 10^−3^ substitutions per site per year, we would expect approximately 22 nucleotide substitutions over a 9- to 10-month period.

The ratio of non-synonymous substitutions (dN) and synonymous substitutions (dS) were compared between day 1 and day 12 showing a substitution rate of 0.4323 for the NP gene, 0.6717 for the VP35 gene, 0.0961 for the GP gene, and 0.3545 for the L gene, with VP40, VP30 and VP24 showing no evidence of nucleotide substitution. This shows potentially differential selection pressure acting along the EBOV genome during infection. The analysis was similarly performed between day 9 (when we postulate EBOV became established in a state of less active replication) and the samples taken in 2015 using yn00 in the paml package. Unlike the small rate of substitution seen between days 1 and 12, comparison between day 9 and recrudescent infection several months in 2015 later showed no significant genomic changes.

## Discussion

Whilst both the host response [6, 7, 30] and the presence of co-infections [8] may contribute to the outcome in EVD, the overwhelming factor is viral load, with high viral loads (Ct < 18.1) being associated with a fatal outcome [4, 5]. The analysis of both viral load and the host response through sequencing blood samples from UK2 and comparison to equivalent matched patients from Guinea revealed the severity of the infection in UK2 (Table 1). In our experience, West African patients with these viral loads invariably had a fatal outcome. Similarly, in West African patients upon testing at the Ebola Treatment Centre, in those patients who succumbed to the disease, there was a greater upregulation of interferon signalling and acute phase responses, compared to patients who went on to survive infection [6]. Particularly notable was the strong upregulation of gene transcripts associated with significant liver pathology. Cell subtype prediction using mRNA expression patterns in the blood indicated that natural killer (NK) cell populations were greater in patients who went on to survive infection versus patients who went on to have a fatal outcome [6]. Fatal EVD in these patients was also characterised by strong T-cell activation [34]. The data indicated that the abundance of transcripts associated with severe and fatal infection in West African patients [6] at the early time points in UK2, e.g. days 5 and 6, was greater (Figs. 2 and 3), suggesting that UK2 would have been predicted to have a fatal outcome. This frames the question of how UK2 survived.

UK2 was administered a monoclonal antibody cocktail, ZMab, on two occasions. Data from experiments in non-human primate models of EVD suggested that such therapeutics enhanced the chance of survival. For example, the monoclonal antibody cocktail MB-003, provided 50–100% survival in rhesus monkeys when administered within 24–72 h after EBOV inoculation [41]. A different cocktail of antibodies called ZMAb (Public Health Agency of Canada) resulted in 100% survival when administered to cynomolgus macaques 24 h after exposure to EBOV [42], and was used to treat a repatriated healthcare worker [43]. During the 2013–2016 EBOV outbreak in West Africa, a different formulation called ZMapp was also used. This had also previously been shown to be effective in the treatment of nonhuman primates with EVD after 5 days post experimental infection [44]. Although a randomised control trial in humans using a combination of ZMapp plus the current standard of care versus the current standard of care alone showed some benefit, the study did not reach the prespecified statistical threshold for efficacy [11]. In certain examples, EBOV escape mutants have been identified when antibody-based therapy has been used to inhibit/reduce EBOV infection in non-human primate models of infection [45]. The RNAseq data from UK2 revealed a decrease in reads mapping to the viral genome after the application of the monoclonal antibody cocktail ZMab. This was perhaps a more accurate indication of the amount of virus than the RT-qPCR analysis. Although UK2 was effectively a N of one, we hypothesise that given both viral and cellular markers were predictive of a fatal outcome, the application of ZMab was responsible for the dramatic lowering of viral load, and the change in the patient from a predicted fatal outcome to a survival pathway. This was perhaps evidenced by the DCQ analysis suggesting a greater abundance of natural killer cells as the time increased post-presentation and viral load decreased (Fig. 4). DCQ analysis of samples from West African survivors at the acute phase of infection suggested a greater abundance of NK cells compared to equivalent patients who went on to have a fatal infection [6]. This was confirmed in further experiments using flow cytometry [46], where CD56 negative NK cells were associated with a survivor outcome [46]. The DCQ analysis of the samples from UK2 predicted an increase in CD56- NK cells (Fig. 4).

Given the scale of the outbreak and attention, several aspects of EVD came to the forefront including the potential for recrudescence after the initial infection and persistence of the virus in immune privileged sites [13, 14, 16]. Similar to patients from West Africa and other repatriated health care workers [14], UK2 suffered a recrudescent infection 10 months after their initial infection—despite there being no evidence of EBOV in the blood after the first infection. Several mechanisms may account for the recrudescence, including a ‘latent state’ and the possible role of DIs as way of modulating virus replication [39] to levels below the limit of detection. Certainly, this hypothesis is not without merit, as defective measles virus genomes have been identified in the brain cells of patients with subacute sclerosing panencephalitis (SSPE) [47]. If this were the case with UK2, we would hypothesise that DIs would compete for viral proteins involved in RNA synthesis and other processes and maintain low levels of viral genomes. Recrudescence would then be explained through the resurgence of the viral genome—similar to predator/prey relationships. Analysis of the RNA sequencing data from both the initial infection and recrudescence in UK2 indicated that at no point did the DIs become the majority population. Overall, we conclude that DIs (assuming they are not artefacts of the sequencing protocols) may have been present at levels where they did not outcompete genomic replication and therefore did not significantly impact virus lifecycle.

Rather, based on the lack of viral sequence diversity/evolution between the initial infection and relapse, we favour a model of the virus entering a state of reduced virus replication, as suggested by the phylogenetic analysis based on coding versus non-coding changes (Fig. 2). Recently, models of persistence in RNA viruses that cause acute infections have been proposed where single amino acid substitutions in viral proteins can result in aberrant RNA replication [40]. Restoration of the wild-type or alternative functional amino acid results in lytic infection [40]. To account for the recrudescent infection in UK2, we hypothesise that whilst a robust adaptive immune response cleared the consensus EBOV genome population in the original infection, minor variant virus populations were harboured in immune privileged sites.

These minor variants may have been deficient in replication and protected from the immune response. This would lead to persistence of the virus. In which case, the restoration of efficient replication through mutation and random chance led to a second EBOV infection and potentially the requirement for the generation of further neutralising antibodies. Analysis of the virus population genetics in the initial infected revealed a narrowing of the minor variants from day 4 to day 10. To account for this observation, we postulated that although the immune system will be directed against the whole virus population, the dominant immune response would have been against the consensus phenotype/genotype of the virus, potentially eliminating the consensus genotype viruses at an accelerated rate compared to viruses carrying minor variants. Analysis of the minor variant frequency in the samples taken during recrudescence indicated fewer variants compared to the original infection.

## Conclusions

This sequencing analysis provided a longitudinal natural history of infection and the corresponding host response in a patient with EVD. Together with the supportive care, the use of the medical countermeasure ZMab was likely to have changed UK2’s pathway from a fatal outcome to survival. The study thus provides direct molecular evidence to support the supposition by one of the authors (and attending physician for UK2) [17] that despite an initial unfavourable prognosis (i.e. death), disease management and treatment in extreme cases of EVD are worth pursuing.

## Data Availability

All raw sequencing reads from this study have been deposited under NCBI project PRJNA668038 BioProject (https://www.ncbi.nlm.nih.gov/bioproject/PRJNA668038) [48].
